# Glycolipids produced by *Rouxiella* sp. DSM 100043 and isolation of the biosurfactants via foam-fractionation

**DOI:** 10.1186/s13568-015-0167-7

**Published:** 2015-12-23

**Authors:** Johannes H. Kügler, Claudia Muhle-Goll, Silla H. Hansen, Annika R. Völp, Frank Kirschhöfer, Boris Kühl, Gerald Brenner-Weiss, Burkhard Luy, Christoph Syldatk, Rudolf Hausmann

**Affiliations:** Section II: Technical Biology, Institute of Process Engineering in Life Sciences, Karlsruhe Institute of Technology, Engler-Bunte Ring 1, 76131 Karlsruhe, Germany; Institute of Organic Chemistry, Karlsruhe Institute of Technology, Fritz-Haber Weg 6, 76131 Karlsruhe, Germany; Institute for Biological Interfaces 1, Karlsruhe Institute of Technology, Hermann-von-Helmholtz-Platz 1, 76344 Eggenstein-Leopoldshafen, Germany; Section Applied Mechanics, Institute for Mechanical Process Engineering and Mechanics, Karlsruhe Institute of Technology, Gotthard-Franz-Strasse 3, 76131 Karlsruhe, Germany; Department Microbiology of Natural and Technical Interfaces, Institute of Functional Interfaces, Karlsruhe Institute of Technology, Hermann-von-Helmholtz-Platz 1, 76344 Eggenstein–Leopoldshafen, Germany; Institute for Biological Interfaces 4, Karlsruhe Institute of Technology, Hermann-von-Helmholtz-Platz 1, 76344 Eggenstein-Leopoldshafen, Germany; Section Bioprocess Engineering, Institute of Food Science and Biotechnology, University of Hohenheim, Garbenstr. 25, 70599 Stuttgart, Germany

**Keywords:** Biosurfactant, Surfactant, Emulsifier, Glycolipid, *Rouxiella*, *Serratia*, Talose, Hydroxy linoleic acid, Myristic acid, Myristoleic acid

## Abstract

Microorganisms produce a great variety of secondary metabolites that feature surface active and bioactive properties. Those possessing an amphiphilc molecular structure are also termed biosurfactant and are of great interest due to their often unique properties. *Rouxiella* sp. DSM 100043 is a gram negative enterobacter isolated from peat-bog soil and described as a new biosurfactant producing species in this study. *Rouxiella* sp. produces glycolipids, biosurfactants with a carbohydrate moiety in its structure. This study characterizes the composition of glycolipids with different hydrophobicities that have been produced during cultivation in a bioreactor and been extracted and purified from separated foam. Using two dimensional nuclear magnetic resonance spectroscopy, the hydrophilic moieties are elucidated as glucose with various acylation sites and as talose within the most polar glycolipids. The presence of 3′ hydroxy lauroleic acid as well as myristic and myristoleic acid has been detected.

**Electronic supplementary material:**

The online version of this article (doi:10.1186/s13568-015-0167-7) contains supplementary material, which is available to authorized users.

## Introduction

A great variety of surfactants occur as metabolites synthesized by various microorganisms. Their structures are versatile and many different hydrophilic and hydrophobic moieties are described in literature (Hausmann and Syldatk 2014; Kügler et al. 2015). Within glycolipids, the hydrophilic moieties usually are composed of one or more sugar components, mainly present in their ring form. Glucose, sophorose, rhamnose, mannose and the disaccharid trehalose are best studied as hydrophilic moieties of glycolipid biosurfactants. Linked to these are a variety of different lipophilic moieties, largely described are fatty acids of variable length.

Microbial surfactants can, besides differences in their structural composition, as well be different in their physiological characteristics such as foaming. The formation of foam that builds up pressure in bioreactors is a challenge within the production of biosurfactants. Foam-fractionation, the separation of foam via an outlet of the reactor during the cultivation process has been successfully applied within the production of biosurfactants (Chen et al. 2006; Davis et al. 2001; Willenbacher et al. 2014) and not only hinders the increase of pressure in the reactor but also displays a first step of product removal.

The strain *Rouxiella* sp. DSM 100043 is an isolate of the upper layer of a pristine raised bog, a habitat rich in carbon sources such as humic substances but deficient in other nutrients. The production of surface active or emulsifying compounds as secondary metabolites and the release of enzymes envolved may serve as a tool for an accession of nutrients, swarming, or defense of habitat and displays an adaption to living conditions in the acrotelm of peat-bog areas.

This study characterises amphiphiles produced by *Rouxiella* sp. DSM 100043, describes a production method using glycerol as carbon source as well as the extraction and purification of glycolipids from fractionated foam. The utilization of two dimensional NMR septroscopy is used for structural characterization of the glycolipids produced.

## Materials and methods

### Microorganism

Peat was sampled in a raised bog of the northern Black Forest near Kaltenbronn, Germany (48.719°N, 8.459°E) at a depth of approximately 2–5 cm and stored at −20 °C. The soil was resuspended in sterile demineralised water and dilutions were streaked onto yeast-malt (YM) agar plates containing per liter: 3 g yeast extract, 3 g malt extract, 5 g peptone, 10 g glucose and 20 g agar set to a pH of 7.0 using NaOH and/or H_3_PO_4_. Agar plates were incubated at 20 °C until colonies were clearly visible. Morphological different colony forming units (*cfu*) were picked with a sterile tip and streaked out onto fresh YM agar plates, repeatedly grown and picked for at least three times or until visible purity. Isolates were stored in cryo-stocks at −80 °C in YM containing 15 % glycerol and used as inoculate for all experiments. The isolated strain *Rouxiella* sp. 323 was submitted to the German Collection of Microorganisms and Cell Cultures (DSMZ, Braunschweig, Germany) and assigned as *Rouxiella* sp. DSM 100043.

Gram characteristics of the strain was determined by mixing a drop of 3 % potassium hydroxide with a loop of a single colony on a glass surface by stirring with a needle for 1 min. Bacteria were determined to be gram negative when the formation of threads was observed after lifting the stirring device.

Phylogenetic affiliation was determined genetically. 10 ml YM overnight cultures from a single colony of *Rouxiella* sp. DSM 100043 was centrifuged for 20 min at 4643×*g* and 4 °C. The genomic DNA of each cell pellet was extracted using PureLink Genomic DNA Mini Kit (Life Technologies GmbH, Darmstadt, Germany) according to the supplier’s manual. Genomic DNA (gDNA) was eluted in nuclease free water (Carl Roth GmbH, Karlsruhe, Germany). The 16S rRNA decoding DNA sequence was amplified by polymerase chain reaction (PCR) containing 5 μl of 1:10 diluted DNA template in nuclease free water, 0.75 U polymerase (HotStar TaqTM, Qiagen, Hilden, Germany), 0.6 μl desoxyribonucleotide triphosphate mix (dNTPs; 10 mM of each dNTP: Qiagen, Hilden, Germany), 1 μl of oligonucleotide 27F (100 pmol µl^−1^; 5′-AGAGTTTGATCCTGGCTCAG-3′) and 1 µl of oligonucleotide 1385R (100 pmol µl^−1^; 5′-CGGTGTGTRCAAGGCCC-3′ whereas R is A or G) (both Biomers, Ulm, Germany), and 3 µl of a PCR reaction buffer (10×, Qiagen, Hilden, Germany) filled up to a total volume of 25 µl per sample with nuclease free water. Reaction took place in a thermocycler (Master Cycler Gradient, Eppendorf, Hamburg, Germany) programmed as follows: single activation step 15 min at 95 °C followed of 30 cycles comprising: (1) initial denaturation 1 min at 94 °C, (2) annealing 1 min at 55 °C, (3) elongation 1 min at 72 °C, followed by a terminating elongation step for 10 min at 72 °C with a subsequent storage temperature of 4 °C. Amplification of DNA was checked by gel electrophoresis. 5 µl of each sample was mixed with 1 µl loading dye and loaded onto a roti-safe (Carl Roth GmbH, Karlsruhe, Germany) stained 1 % agarose gel in tris base boric acid EDTA buffer (TBE; containing per liter 10.8 g tris base, 5.5 g boric acid, 20 mM EDTA) and migrated for approximately 1 h at 90 V. For visualizing DNA fragments the gel was irradiated with 312 nm UV light and the size of the amplified ~ 1.4 kb sized fragments was compared with a co-migrated 0.1–10 kb DNA ladder (QuickLoad 2-log, New England Biolabs, Frankfurt/Main, Germany). DNA fragments were sequenced (GATC, Konstanz, Germany) from both sides, submerged and after exclusion of each ends flanking 30 base pairs compared with the 16S rRNA sequences of culturable species using the National Center for Biotechnology Information MEGABLAST tool and databse (http://blast.ncbi.nlm.nih.gov/Blast.cgi). Mismatches to the sequences of the most similar type strains were checked manually in the sequence spectrograms. Sequences were checked for chimeras using DECIPHER search tool (Wright et al. 2012). 16S rRNA sequence of *Rouxiella* sp. DSM 100043.was submitted to NCBI GenBank [GenBank: KP642161].

### Production

For the production of glycolipids a glycerol basal media (GBM3) adapted from Roldán-Carrillo et al. (2011) was used containing per litre: 3.0 g (NH_4_)_2_SO_4_, 50 g glycerol, 1.1 g KCl, 1.1 g NaCl, 1.0 g MgSO_4_, 2.33 mg FeSO_4_·7H_2_O and a phosphate buffer of 4.4 g K_2_HPO_4_, 3.4 g KH_2_PO_4_ for shake flask experiment respectively 1.1 g K_2_HPO_4_, 0.85 g KH_2_PO_4_ for bioreactor cultivation. The medium was enriched with 5 ml of a trace element solution containing per litre 0.29 g ZnSO_4_·7H_2_O, 0.19 g CaCl_2_·2H_2_O, 0.25 g CUSO_4_·5H_2_O, 0.17 g MnSO_4_·7H_2_O. 20 µl of *Rouxiella* sp. DSM 100043 cryo-stock were inoculated in 20 ml GBM3 (100 ml baffled conical flasks) and cells were grown over night at 30 °C and 130 rpm then transferred into 100 ml GBM3 (1000 ml baffled conical flasks) and again grown for approximately 35 h until an optical density at λ = 600 nm (OD_600_) of 6 was reached.

For batch fermentation stirred 2.5 l bench-scale bioreactors (Minifors, Infors, Bottmingen, Switzerland) were used, each equipped with a double foam trap consisting of switchable single use bags for foam fractionation. Bioreactors were filled with GBM3 media and inoculated from shake flasks to a starting optical density of 0.1 (OD_600_) in an operating volume of 1 l. The processes were run for approximately 75 h at a controlled temperature of 30 °C. Physiological activities were monitored by internal pO_2_ electrodes and pH 7.0 was controlled and adjusted by internal pH-electrodes using 4 M H_3_PO_4_ and 4 M NaOH. Airflow of 0.1 vvm was kept constant throughout the process; dissolved oxygen was maintained between 8 and 18 % by varying stirring speeds between 300 and 1200 rpm. The fermentation process was controlled and recorded using a bioprocess software (Iris 5, Infors, Bottmingen, Switzerland). Foam formed during the cultivation process was collected via the exhaust cooler in single use bags that were cooled on ice to prevent further growth of foamed microorganisms.

During the fermentation processes, 12 ml culture samples were taken as duplicates at different time points for the analysis of growth characteristics. Optical density was determined using concentration dependent dilutions with 0.9 % (w/v) NaCl. For gravimetric determination of dry cell mass 10 ml of the culture broth from each sampling point was transferred into dry-weighed 15 ml sampling tubes and centrifuged for 20 min at 4643×*g* and 4 °C. The supernatant of each sample was transferred into a new tube and stored at −20 °C prior to the determination of ammonia ions, glycerol content and surface tension. The remaining cell pellet was washed with 5 ml 0.9 % (w/v) NaCl followed by centrifugation (10 min at 4643×*g* and 4 °C), decanting and drying to constant weight in a drying closet at 100 °C.

Foam bags were replaced five times during the process, liquid and foam from each bag was wringed into weighed 50 ml sampling tubes and centrifuged at 4643×*g* and 4 °C for 20 min or until foam was fluidified. Spun down cell masses were carefully solubilised and OD_600_ was measured with adequate dilutions in 0.9 % (w/v) NaCl. Samples were again centrifuged (14,000 rpm, 20 min, 4 °C), the supernatant transferred into a fresh tube and stored at −20 °C. For gravimetric determination of dry cell mass in the foam fractions, the remaining cell pellets were washed and dried until constant weight as described.

The ammonium ion concentration in the supernatant was determined by an ammonia assay using photometric quantification (Spectroquant 109713, Merck, Darmstadt, Germany) downscaled to a fifth of the volumes listed in the supplier’s manual, spectrophotometric measurements were conducted in a microtiter plate and concentrations were determined using an ammonia ion standard curve.

Glycerol content in the supernatant was determined using a nicotinamide adenine dinucleotide (NAD^+^) coupled enzymatic test kit with photometric quantification (Boehringer-Mannheim/R-Biopharm, Darmstadt, Germany) by downscaling to a twentieth proportion of the volumes listed in the supplier’s manual and quantification via glycerol standard curves in a microtiter plate.

Dry cell mass, glycerol and ammonia ion data points were fitted (SigmaPlot, version 12.5, Systat Software, Inc., Washington, USA) using a logistic model with four parameters (Zwietering et al. 1990). All fermentation results are plotted as mean values of two fermentation processes with each data point measured as duplicate for dry cell mass and triplicate for glycerol content and ammonia ion concentration. Alteration in the surface tension of samples taken from the fermentation supernatant as well as of foam trap samples were monitored against air at room temperature using the Du Noüy (1919) ring method on a Tensiometer (Lauda TD1, Lauda-Königshofen, Germany) according to the supplier’s manual. Trend of the surface tension values in the bioreactor was fitted using a linear equation.

### Extraction and isolation of glycolipids from foam

Supernatants from the fluidifized foam were acidified until neutral pH using H_3_PO_4_ and subsequently extracted twice using 1.25 volumes of ethyl acetate (v/v) in 12 ml screw cap glass vials with subsequent centrifugation (10 min at 4643×*g*, 4 °C). The combined organic phases were concentrated using a rotary evaporator (Heidolph Laborota 4000, Schwabach, Germany) at 40 °C and 240 mbar followed by vacuum concentration at 40 °C, 2000 rpm and 50 mbar (ScanSpeed MiniVac Evaporator, Saur, Reutlingen, Germany) to gain crude extract. Qualitative detection of the glycolipids was performed by thin layer chromatography (TLC) using 60 Å silica TLC plates (Alugram Xtra SIL G, Macharey-Nagel, Düren, Germany) as stationary phase and a mobile phase of isopropyl acetate/methanol/acetic acid (100:10:1 v/v/v). Glycolipids and fatty acids were detected by dipping the TLC plate into 10 % (v/v) H_2_SO_4_ and development under 180 °C air stream for 4–5 min.

The crude extract was dissolved in two times 20 ml 10 % (v/v) methanol in ultrapure water and further purified for structural analysis by medium-pressure-liquid-chromatography (MPLC; SepacoreX50, Büchi, Flawil, Switzerland) using prepacked 40–63 µm particle size reverse phase C18ec columns (RP18ec; 150 mm column length, 12 mm column diameter and 17 ml bed volume; Büchi, Flawil, Switzerland) with a ultrapure H_2_O / methanol gradient solvent system for 90 min at a flow rate of 10 ml min^−1^ (gradient: 15 min 100–100 % H2O; 45 min 100–0 % H_2_O; 30 min 0–0 % H_2_O). The eluate was collected in 10 ml fractions. From each separation fractions 60–61, 64–65 and 67–69 were combined and the solvent was evaporated, the sample lyophilized (Beta 2-16, Martin Christ GmbH, Osterode, Germany) and used for structural analysis of the fatty acids. Fractions containing the glycolipids (63–65) were combined and again purified to remove residual fatty acids before structural analysis.

To further elucidate the sugarsystems, the fraction was dissolved in 20 ml isopropyl acetate/methanol (1:1 v/v) and further purified using 40–63 µm particle size silica stationary phase with 60 Å pore size (150 mm column length, 12 mm column diameter and 17 ml bed volume; Büchi, Flawil, Switzerland) and manually eluted isocratically with isopropyl acetate/methanol (24/1 v/v). The eluate was collected in 10 ml fractions, fraction 2–3 contained fatty acids, the other fractions were combined to samples 63-65 A (fractions 4–6), 63-65 B (fraction 7), 63-65 C (fractions 8–10), 63-65 D (fractions 11–16) and 63-65 E (fractions 17–23), the solvent evaporated and the samples again lyophilized.

### Structural analysis

For nuclear magnetic resonance (NMR) spectroscopy fractions containing the fatty acids (60–61 and 67–69) as well the glycolipids (64–65 and subfraction 63–65 E) were dissolved in 0.6 ml CDCl_3_/CD_3_OD (both 7:3 v/v) (Sigma-Aldrich; Germany). One dimensional ^1^H NMR spectroscopy and two dimensional ^1^H–^1^H correlation spectroscopy (COSY), total correlation spectroscopy (TOCSY), nuclear Overhauser enhancement spectroscopy (NOESY), ^1^H–^13^C heteronuclear single quantum coherence spectroscopy (HSQC) and heteronuclear multiple-bond correlation spectroscopy (HMBC) were recorded on a Bruker AVANCE II + 600 MHz spectrometer (Bruker AG, Rheinstetten, Germany) equipped with a BBI probe head. Spectra were analyzed with Topspin 3.2 (Bruker AG) and Spinworks 3.1.8 software (Marat, University of Manitoba, USA). Intensities were measured from a one dimensional ^1^H spectrum acquired with a single scan. Chemical shifts are referenced to the ^1^H and ^13^C resonance of the residual CHCl_3_ signal.

Mass determination of subfractions 63–65 A to 63–65 E was performed using ESI-Q-ToF (Q-Star Pulsar, AB SCIEX, Darmstadt, Germany). Small amounts of dried fractions were dissolved in methanol/H_2_O/acetic acid (v/v/v 500:500:1) containing 5 ppm LiCl. Samples were continuously infused via a syringe pump at a flow of 10 µl min^−1^. The system was operated in the positive mode with a heater temperature of 300 °C. The spray tip voltage was set to 5000 V, the declustering potential was 30 V and the focusing potential was 60 V. The Nebulizer gas and the curtain gas was nitrogen 5.0. Spectra were recorded in in a mass range from m/z 50 to m/z 1200 in the activated “enhance all” mode at an accumulation time of 1 s. The ESI-Q-ToF was calibrated using a calibration standard (M600, Applied Biosystems) and the measuring accuracy was determined to be ±0.05.

## Results

The strain *Rouxiella* sp. DSM 100043 has been chosen for the production of glycolipids due to results in screening experiments, novelty of the genera in terms of biosurfactant production and the product portfolio revealed using functional staining in TLC. According to its 16S rRNA coding sequence strain *Rouxiella* sp. DSM 100043 was, besides the genus *Rouxiella* in close proximity to a range of other enterobacteria such as *Serratia*, *Rahnella*, *Yersinia*, *Ewingella* and *Hafnia* spp. The isolated strain did not show growth at 37 °C, as also reported for its closest 16S rRNA relative (Le Fleche-Mateos et al. 2015) and was therefore assigned to the genus *Rouxiella* and submitted as *Rouxiella* sp. DSM 100043.

**Fig. 1:**
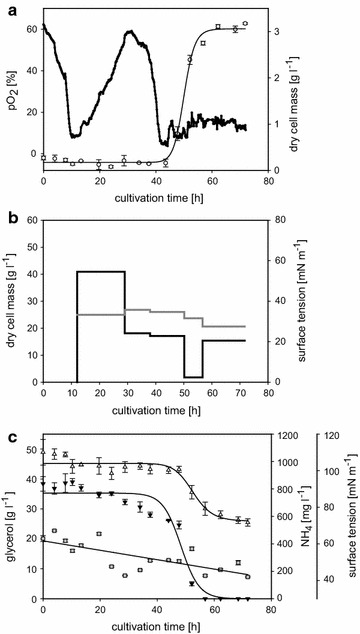
Growth parameters of *Rouxiella* sp. DSM 100043 during biosurfactant production. Time course of **a** dissolved oxygen (*solid line*) and dry cell mass (*open circle*) in the bioreactor system and **b** of dry cell mass (*black line*) and surface tension (*grey line*) examined from fractionated foam. **c** Time course of the surface tension (*grey square*) in the reactor as well as depletion of glycerol (*open triangle*) and ammonium (*filled inverted triangle*)

Glycolipids of strain *Rouxiella* sp. DSM 100043 were produced as duplicates in 1 litre bench scale bioreactors and fractionated in foam traps during the cultivation. The fermentation processes took place under nitrogen limiting conditions in a mineral medium with glycerol as carbon source; glycolipids were extracted from fluidifized foam trapped in the foam bags. Figure 1a, c show physiological conditions present within the bioreactor system. Remote consumption of carbon and nitrogen within the first 10 h of cultivation, as well as a decrease of dissolved oxygen indicates growth of *Rouxiella* sp. in the beginning of the fermentation. An increase of optical density and dry cell mass in the bioreactor (Fig. 1a) was not observed within this time period. The formation of foam started about 2 h after inoculation, filling up the reactor void and exiting via the exhaust gas cooler until being captured in the foam bag traps after 10 h of cultivation (Fig. 1b). Between 10 and 40 h of cultivation, nutrients were consumed steadily and biomass formed continuously led into the foam traps where a concentration of up to 40 g l^−1^ dry cell mass was reached. Between hours 40–60, growth of *Rouxiella* sp. approaches an exponential phase, so is the decrease of carbon and nitrogen concentrations in the cultivation media (Fig. 1c). With low pO_2_ values reached after 60 h of cultivation an increase of stirring speed was regulated and cells accumulate in the cultivation media (Fig. 1a). The foaming off of cells decreases during that period (Fig. 1c). Surface tensions in supernatants from the reactor medium are fluctuating with a decreasing trend indicated by fitting of the data but remain above 40 mN m^−1^ throughout the process (Fig. 1c). Surface tension values steadily remained below 28 mN m^−1^ in fluidifized foam collected from all traps (Fig. 1b).

**Fig. 2:**
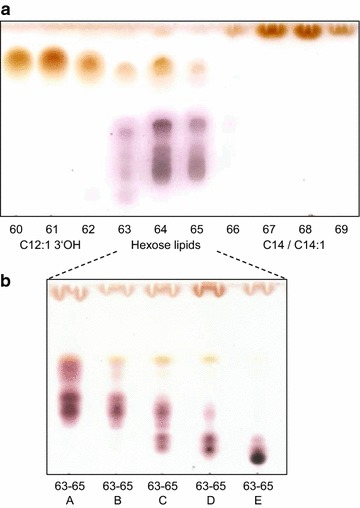
Thin layer chromatography (TLC) of glycolipids extracted and purified from foam fractionated during cultivation. TLC plates are stained with sulphuric acid. **a** 3′ hydroxyl lauroleic acid present in fractions 60–62, glycolipids present in fractions 63–65 and myristic as well as myristoleic acids present in fractions 67–69. **b** TLC of further purified glycolipids from fraction 63–65 resulting in subfractions 63–65 A to 63–65 E with the most hydrophilic glycolipids in 63–65 E containing talose as carbohydrate moieties

**Fig. 3:**
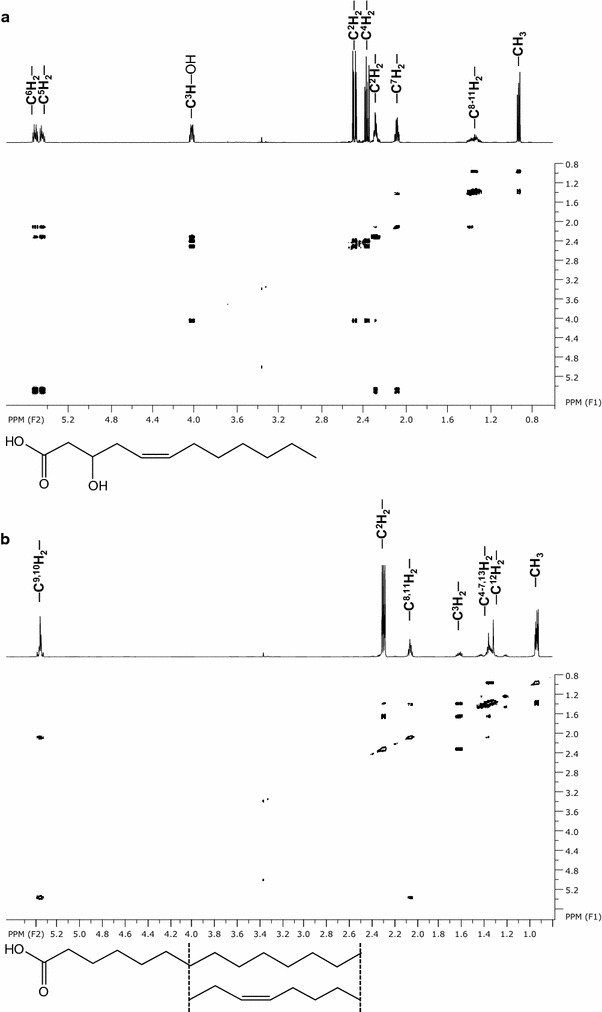
Assigned spectra of two dimensional ^1^H/^1^H correlated spectroscopy (COSY). **a** Derived from fractions 60–61 elucidated as 3′ hydroxy lauroleic acid and **b** derived from fractions 67–69 elucidated as a mixture of myristic and myristoleic acids. Both are shown as molecular structures

A total of 145 ml fluidifized foam was collected per batch cultivation with a total cultivation volume of 1 l. Glycolipids as well as fatty acids were detected in the foam that with pH 9 had a relatively alkaline character. The pH was neutralized prior to the extraction and purification of the components. 119.2 mg l^−1^ crude extract was yielded per batch process after triple extraction of the fluidifized foam. TLC and subsequent staining of the extracts revealed the presence of two different fatty acid molecules as well as a mixture of glycolipids that varies within their retardation factors. Reverse-phase chromatographic separation of 238.4 mg extract from both fermentations allowed an isolation of the fatty acids and the glycolipids (Fig. 2a). The total yield of the fractions after purification steps was 19.9 mg of fractions 60–61, 27.9 mg of fractions 64–65 and 34.9 mg of fractions 67–69. The fatty acids could be unambiguously elucidated from the pattern of ^1^H COSY, ^13^C HSQC and ^13^C HMBC as 3′ hydroxyl lauroleic acid for the more hydrophilic fractions 60–61. Fractions 67–69 most probably contained a mixture of myristic and myristoleic acids deduced from ^1^H COSY (Fig. 3; Table 1), ^13^C HSQC and ^13^C HMBC NMR spectroscopy and the ratio of intensities for C^x^H_2_, C^ω^H_3_, C^2^H_2_ and C^3^H_2_ in ^1^H 1D NMR spectrum.

**Table 1:** NMR data of fatty acids moieties

	C Shift [ppm]	H Shift [ppm]	Multiplicity	Coupling [Hz]
3′ OH C12:1 FA				
O=C^1^–OH	175.95			
–C^2^H_2_–	42.39	2.46, 2.24	dd, dd	15.4, 8.25, 4.6
–C^3^H–OH	69.6	4.00	m	
–C^4^H_2_–	35.8	2.26	m	
–C^5^H=	125.7	5.43	m	
–C^6^H=	133.5	5.50	m	
–C^7^H_2_–	28.3	2.05	q	7.1
–C^8^H_2_–	30.7	1.37	m	
–C^9^H_2_–	30.1	1.34	m	
–C^10^H_2_–	32.9	1.31	m	
–C^11^H_2_–	23.7	1.33	m	
–C^12^H_3_	14.4	0.90	t	6.8
C14/C14:1 FA				
O=C^1^–OH	177.8			
–C^2^H_2_–	35.1	2.27	t	7.4
–C^3^H_2_–	26.1	1.59	m	
–C^4^H_2_–	30.26	1.33		
–C^5^H_2_–	30.6	1.33		
–C^6^H_2_–	30.3	1.33		
–C^7^H_2_–	31.1	1.33	m	
–C^8^H_2_–	28.17	2.03	dd	12.3, 6.4
–C^9^H_2_–/–C^9^H=	130.9	5.34		
–C^10^H_2_–/–C^10^H=	130.9	5.34		
–C^11^H_2_–	28.17	2.03	dd	12.3, 6.4
–C^12^H_2_–	33.1	1.29	m	
–C^13^H_2_–	23.8	1.32	m	
–C^14^H_3_	14.6	0.89	t	6.6

Chemical shifts of carbon and hydrogen nuclei, multiplicity of the peak observed and its coupling constant of 3′ hydroxy lauroleic acid from fractions 60–61 (3′OH C12:1 FA) and potential myristic/myristoleic acid from farctions 67–69 (C14/C14:1 FA)

*FA* fatty acid, *d* doublet, *t* triplet, *q* quartett, *m* multiplet

**Fig. 4:**
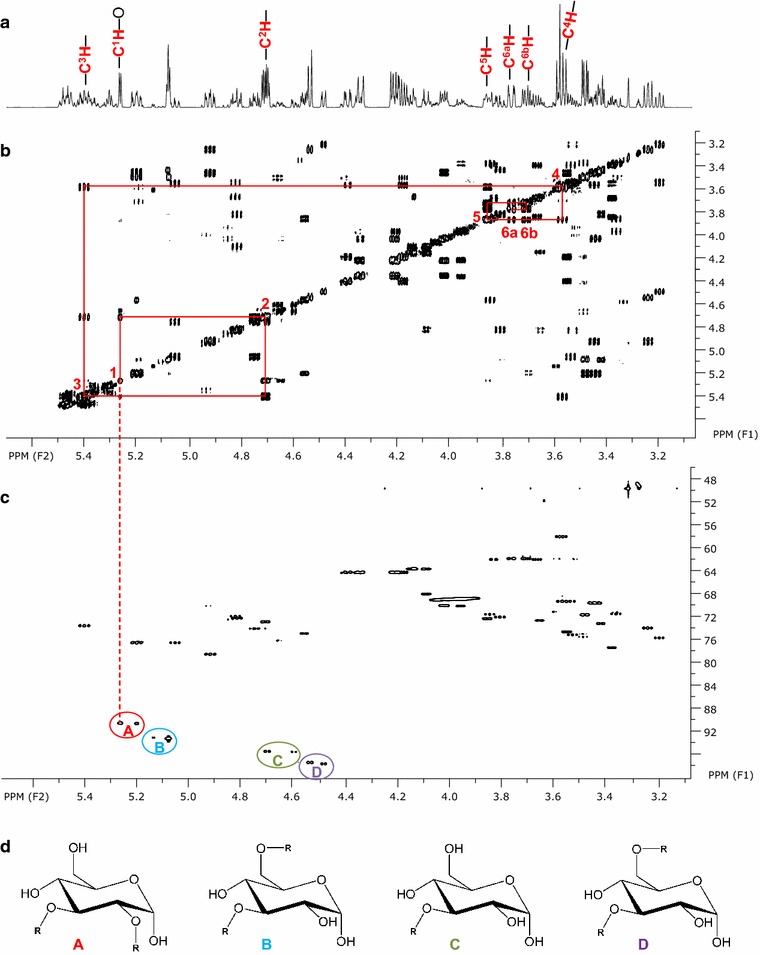
NMR spectra of *Rouxiella* sp. DSM 100043 glycolipids present in fractions 64–65. Close-up of the carbohydrate region is shown: **a** ^1^H spectrum, **b** two dimensional ^1^H/^1^H COSY and **c** two dimensional ^1^H/^13^C HSQC spectrum. Anomeric C1 of the glucose moieties A to D in both, α and β configuration is shown in **c**, molecular structures in **d**. The more dominant form of sugar A, carrying acylation at C2 and C3 is exemplarily assigned in red in **a** ^1^H spectrum and **b** as *red lines* in the COSY spectrum

The mixture of glycolipids present in the combined fractions 64–65 (Fig. 2a) contained as hydrophilic moieties at least four different systems assigned as sugar A, B, C and D present in both diastereomers α and β. The sugar systems were assigned starting at the anomeric carbon atoms that are downshifted in ^1^H/^13^C two dimensional HSQC NMR spectra (Fig. 4c). Neighboring carbon atoms of each sugar system were identified by tracing the spin systems in ^1^H COSY and TOCSY spectra (Fig. 4b; Additional file 1: Figure S4). Exchange cross-peaks between the corresponding anomers were determined using NOESY spectra and are also easily identified in the ^1^H, ^13^C-HSQC spectrum (Fig. 4c). Due to their hydrogen coupling constants of mainly larger than ~7.5 or 9 Hz (Table 2), sugar ring protons could be determined to be all axial, which arranges the substituted hydroxy groups in an equatorial form and thus glucose is the dominant sugar in fractions 64–65. The glycolipids have different acylation patterns. The acylation position could be unambiguously determined from ^13^C HMBC spectra. All sugars A–D are acylated at position C3 with additional acylation at C2 for sugar A and C6 for sugar B and sugar D (Table 1; Fig. 4d). Sugar system A in its more dominant form is exemplarily indicated with red lines in 1H and COSY spectra of Fig. 4a, b. The lipophilic moiety could not completely be identified but carries variable double bonds.

**Table 2:** NMR data of sugar moieties

	C Shift [ppm]	H Shift [ppm]	Multiplicity	Coupling [Hz]
Fraction 64–65 (sugar A)				
–C^1^H–O–	91.2	5.33	nd	<1.5
–C^2^H– (acylated C′ 173.1)	73.3	4.80	dd	7.8
–C^3^H– (acylated C′ 173.5)	74.2	5.46	dd	7.8, 9.3
–C^4^H–	69.9	3.613	dd	9.3, 9.6
–C^5^H–	72.5	3.93	m	9.6, 11.9, 5
–C^6a^H–	62.5	3.83	m	12.0, 2.6
–C^6b^H–		3.76	m	12.0, 5.2
Fraction 64–65 (sugar B)				
–C^1^H–O–	93.8	5.17	d	3.9
–C^2^H–	72.1	3.55	nd	>7
–C^3^H– (acylated C’ 174.0)	72.3	5.23	nd	>7
–C^4^H–	73.6	3.51	nd	>7, >7
–C^5^H–	70.4	4.09	dd	10.1
–C^6a^H– (acylated C’ 173.7)	64.8	4.39	m	nd
–C^6b^H–		4.30	m	nd
Fraction 64–65 (sugar C)				
–C^1^H–O–	96.1	4.75	d	7.8
–C^2^H–	74.6	4.83	dd	7.8, >9
–C^3^H– (acylated C’ 173.5)	77.1	5.11	dd	> 9, >9
–C^4^H–	69.7	3.62	dd	> 9
–C^5^H–	77.7	3.42	Overlap	nd
–C^6a^H–	62.6	3.73	Overlap	nd
–C^6b^H–		3.89	Overlap	nd
Fraction 64–65 (sugar D)				
–C^1^H–O–	98.0	4.60	d	7.5
–C^2^H–	74.3	3.33	dd	7.5, >8
–C^3^H– (acylated C’ 173.7)	79.2	4.97	dd	> 9, >9
–C^4^H–	69.9	3.52		>8
–C^5^H–	75.0	3.60		nd
–C^6a^H– (acylated C’ 173.7)	64.8	4.26		nd
–C^6b^H–		4.44		nd
Subfraction 63–65 E (talose)				
–C^1^H–O–	102.1	4.92	d	4.0
–C^2^H–	77.5	4.03	dd (overlap)	4, 4.4
–C^3^H–	76.3	4.26	dd	4.4, 4.4
–C^4^H–	77.1	4.03	dd (overlap)	
–C^5^H–	70.6	3.86	m	7.3, 6.0, 3.7
–C^6a^H–	63.2	3.63	dd	11.5, 6.0
–C^6b^H–	63.2	3.76	dd	11.5, 3.7
Subfraction 63–65 E (acylated talose)				
–C^1^H–O–	109.0	4.76		<1
–C^2^H–	79.7	4.05		<2
–C^3^H–	75.9	4.12		Overlap
–C^4^H–	81.2	4.13		Overlap
–C^5^H–	70.5	3.91	m	
–C^6a^H–	63.8	3.68	dd	11.6, 6.0
–C^6b^H–	63.8	3.82	dd	11.6, 3.3

Chemical shifts of carbon and hydrogen nuclei, multiplicity of the peak observed and its coupling constant from four different glucose lipids (sugar A, B, C and D) present in fraction 64–65, and two talose units present in subfraction 63–65 E. Values are given for the dominant sugar conformations of each sugar moiety

*d* doublet, *m* multiplet, *nd* not determinable

**Fig. 5:**
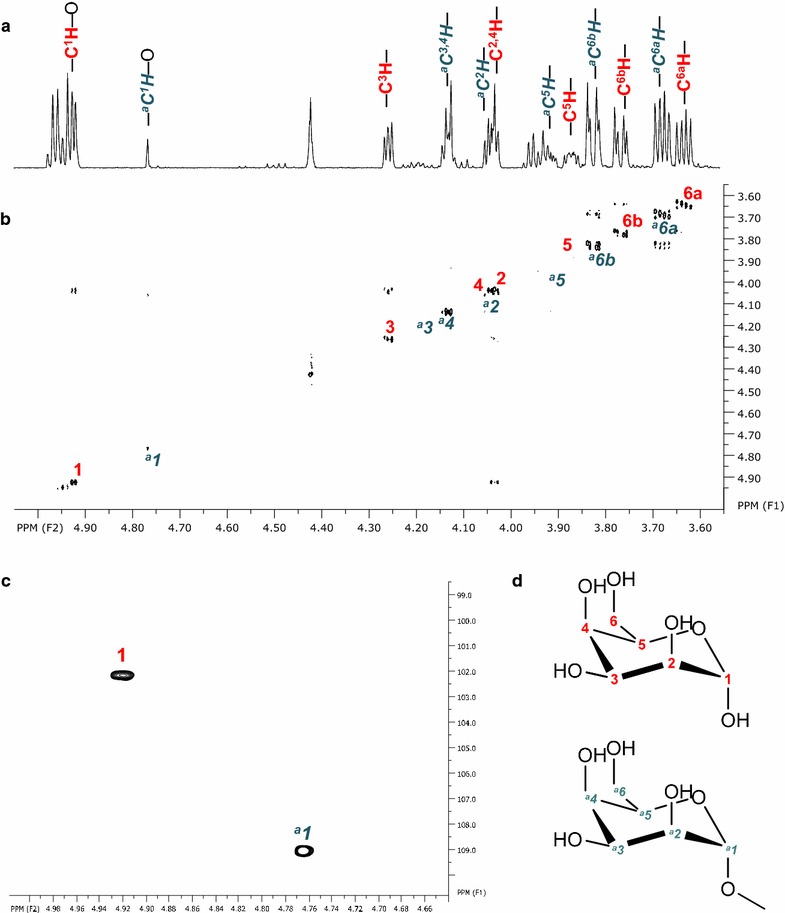
NMR spectra of glycolipids produced by *Rouxiella* sp. DSM 100043 present in subfraction 63–65 E. Close-up of the carbohydrate region in **a** ^1^H spectrum and **b** two dimensional ^1^H/^1^H COSY spectrum and assignment of signals for two sugar moieties C^1–6^ in *red* and ^*a*^ *C* ^*1*−*6*^ in *blue*. **c** Two dimensional ^1^H/^13^C HSQC spectrum revealing two anomeric nuclei: C^1^ and ^*a*^ *C* ^*1*^, the latter downshifted and indicating an acetylation. **d** Potentional molecular structures and assigned C atoms of talopyranose (*red*) and 1′ acetyl talosepyranose (*blue*)

Combined fractions 63–65 were further separated by silica column chromatography into subfractions 63–65 A to 63–65 E that resulted in a partial separation of the glycolipids (Fig. 2b). The concentration in the most hydrophilic subfraction 63–65 E was high enough to conduct two dimensional NMR experiments that revealed two hexose forms as hydrophilic moiety shown by chemical shifts obtained from ^1^H and 2D ^1^H COSY (Fig. 5a, b). According to their coupling constants, these findings mostly refer to the presence of talopyranose (Snyder et al. 1989). The presence of 1′ acetyl-talopyranose as the other sugar moiety in subfraction 63–65 E is indicated by a downshift of the talose C1 nuclei to 109 ppm indicating acetylation at C1 (Fig. 5; Table 2) in ^13^C HMBC and HSQC NMR spectroscopy, respectively.

Mass spectrometric ESI-ToFMS analysis of lithium chloride supplemented subfractions 63–65 A to 63–65 E (see Additional file 1: Table S1 and Figure S1–S3) revealed the presence of both Na^+^ and Li^+^ adduct ions of different *m*/*z* ratios that allowed concluding to the resulting neutral masses present (Additional file 1: Table S1).

## Discussion

*Rouxiella* can be assigned as a new surfactant producing genera. The glycolipid producing strain was identified to be closely related to other enterobacter, such as the genus *Serratia* that hold some known biosurfactant producing species. Examples are *Serratia marcescens* that produces different cyclic lipopeptides (Matsuyama et al. 2011) with antimicrobial, antitumor and plant protecting properties (Thies et al. 2014). Also glycolipids are reported to be produced by *S.* *marcescens* (Dusane et al. 2011) and *S.* *rubidea* (Matsuyama et al. 1990). *Rouxiella* sp. DSM 100043 could be distinguished to relative *Serratia* spp. by limitation in growth temperature. Its inability to grow above 37° C makes this strain unlikely to be pathogenic thus holding advantages as a potential industrial scale biosurfactant producer strain.

Several glycolipids were detected to be produced by *Rouxiella* sp. DSM 100043 in a mineral medium with glycerol as carbon source. The majority of the surface active amphiphiles produced expanded into foam that was formed during cultivation in a bioreactor as indicated by lower surface tension values present in the foam compared to the cultivation medium. Transition of the glycolipids into the foam makes foam-fractionation suitable as a tool for the isolation of the biosurfactants produced.

Reverse phase chromatographic purification of the foam derived extract revealed the presence of three main compound groups as represented in Fig. 2a. Using H_2_O and methanol as solvent system during reverse-phase chromatographic separation, elution of the products took place at high methanol concentrations. Interestingly in the order hydroxyl fatty acid—glycolipid—fatty acid with the most polar gylcolipids as second group and thus not according to their hydrophobicity. A delay of the hexose systems within the elution off the C18 reverse phase column must thus be due to other interactions than hydrophobic.

Two dimensional NMR spectroscopy measurements revealed the presence of 3′ hydroxyl lauroleic acid, and potential myristic as well as myristoleic acid as free fatty acids (Fig. 2; Table 1). The fatty acids revealed in this study are known as common lipophilic moieties of biosurfactants, the shorter hydroxy fatty acid is present in both, glycolipids (Matsuyama et al. 1990) as well as lipopeptides (Matsuyama et al. 2011; Thies et al. 2014) of the relative enterobacteriaceae *Serratia* spp. Glucose moieties with acylations at multiple carbon atoms of the sugar ring are observed (Table 2). It could not be further determined whether an ester or ether bond is present at the acylation site. Mass spectrometric measurements (Additional file 1: Table S1 and Figure S1–S3) hint to the presence of various double bonds that corroborates to the results observed from NMR experiments. Hydrophilic moieties of subfraction 63–65 E revealed the presence of two forms of talopyranose presenting a different hydrophilic moiety to the other glucose lipids present (Fig. 5). Particular for the glycolipids is the absence of an acylation at the anomeric carbon C1 that is usually present in glycolipids (Hausmann and Syldatk 2014; Kügler et al. 2015). It remains unclear whether this absence is a unique property of the glycolipids revealed or due to hydrolysis caused during postprocessing of the glycolipids.

## Additional file

10.1186/s13568-015-0167-7 **Table S1, Figure S1–Figure S3**: Mass spectrometry data and plots of purified foam extracts of Rouxiella sp. DSM 100043. **Figure S4**: Full NMR spectra of Rouxiella sp. DMS 100043 glycolipids present in fractions 64-65.
