# Supplementary material for: Glycolipids produced by Rouxiella sp. DSM 100043 and isolation of the biosurfactants via foam-fractionation
Source: AMB Express. 2015 Dec 23;5:82. doi: 10.1186/s13568-015-0167-7 (PMC4689721; doi:10.1186/s13568-015-0167-7)
Supplement: Supplementary file 1 — 10.1186/s13568-015-0167-7 Table S1, Figure S1–Figure S3: Mass spectrometry data and plots of purified foam extracts of Rouxiella sp. DSM 100043. Figure S4: Full NMR spectra of Rouxiella sp. DMS 100043 glycolipids present in fractions 64-65. [file 13568_2015_167_MOESM1_ESM.pdf]

Additional file

Glycolipids produced by *Rouxiella* sp. DSM 100043 and isolation of the biosurfactants via foam-fractionation

AMB express

Johannes H Kügler[a], Claudia Muhle-Goll[b], Silla H Hansen[a,c], Annika R Völp[a,d], Frank Kirschhöfer[e], Boris Kühl[e], Gerald Brenner-Weiss[e], Burkhard Luy[b,f], Christoph Syldatk[a], Rudolf Hausmann[g]

[a]Institute of Process Engineering in Life Sciences, Section II: Technical Biology, Karlsruhe Institute of Technology, Engler-Bunte Ring 1, 76131 Karlsruhe, Germany

[b]Institute of Organic Chemistry, Karlsruhe Institute of Technology, Fritz-Haber Weg 6, 76131 Karlsruhe, Germany

[c]Institute for Biological Interfaces 1, Karlsruhe Institute of Technology, Hermann-von-Helmholtz-Platz 1, 76344 Eggenstein-Leopoldshafen, Germany

[d]Institute for Mechanical Process Engineering and Mechanics, Section Applied Mechanics, Karlsruhe Institute of Technology, Gotthard-Franz-Strasse 3, 76131 Karlsruhe, Germany

[e]Institute of Functional Interfaces, Department Microbiology of Natural and Technical Interfaces, Karlsruhe Institute of Technology, Hermann-von-Helmholtz-Platz 1, 76344 Eggenstein-Leopoldshafen, Germany

[f]Institute for Biological Interfaces 4, Karlsruhe Institute of Technology, Hermann-von-Helmholtz-Platz 1, 76344 Eggenstein-Leopoldshafen, Germany

[g]Institute of Food Science and Biotechnology, Section Bioprocess Engineering, University of Hohenheim, Garbenstr. 25, 70599 Stuttgart, Germany

Corresponding author:
Johannes H Kügler
Engler-Bunte Ring 1, 76131 Karlsruhe, Germany
+49 721 608 46737
johannes.kuegler@kit.edu

Table S1: Masses present in purified fractions of foam extracts of *Rouxiella* sp. DSM 100043.

Masses observed during ESI-ToF mass spectrometry of lithium chloride supplemented samples in positive mode; assigned lithium $[M_d+Li^+]^+$ and sodium $[M_d+Na^+]^+$ adduct ions led to its resulting neutral mass $[M_{neutral}]$. The strongest relative peak intensity and it's adduct ion type is indicated.

Subfraction	$[M_d + Li^+]^+$	$[M_d + Na^+]^+$	$[M_{neutral}]$	strongest relative peak intensity [%]
**63-65 A**	289.24	305.22	282.23	100 (Li^+)
	256.23	272.21	249.22	56 (Li^+)
	400.26	416.24	393.25	32 (Li^+)
	374.24	390.23	367.23	20 (Li^+)
**63-65 B**	212.19	228.16 / 230.20	205.17 / 207.21	100 (Li^+) / 70 (Na^+)
	238.21 / 240.22	254.18 / 256.21	231.19 / 233.20	67 (Li^+) / 54 (Na^+)
	307.22	323.19	300.20	52 (Li^+)
	271.24	287.22	264.22	33 (Li^+)
**63-65 C**	288.28 / 286.27	304.27 / 302.26	281.28 / 279.27	100 (Li^+) / 40 (Li^+)
	374.24	390.22	367.23	54 (Li^+)
	400.26	416.24	393.25	41 (Li^+)
**63-65 D**	288.28 / 286.27	304.26 / 302.26	281.28 / 279.27	100 (Li^+) / 41 (Li^+)
	213.17	229.14	206.15	56 (Li^+)
	383.23	399.20	376.21	39 (Na^+)
	357.21	373.18	350.19	35 (Na^+)
**63-65 E**	220.09	204.11	197.10	100 (Li^+)
	307.21 / 309.22	323.18 / 325.19	300.19 / 302.20	75 (Na^+) / 23 (Na^+)
	271.24	287.20	264.22	75 (Na^+)

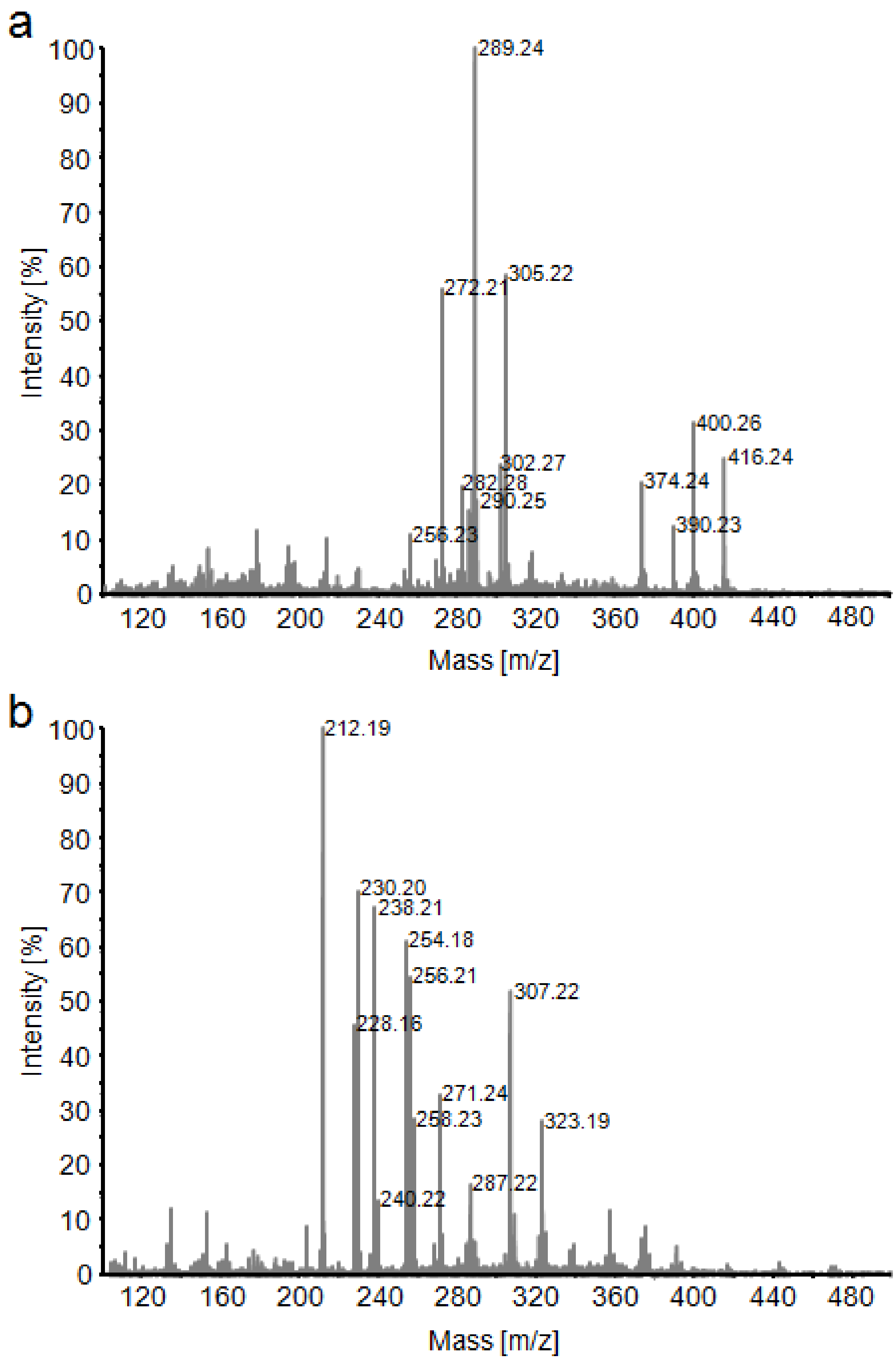

Figure S1: ESI-ToF mass spectrometry plot of purified foam extracts of *Rouxiella* sp. DSM 100043.

Lithium chloride supplemented subfractions (a) 63-65 A and (b) 63-65 B measured in positive mode**.** Both lithium $[M_d+Li^+]^+$ and sodium $[M_d+Na^+]^+$ adduct ions are present

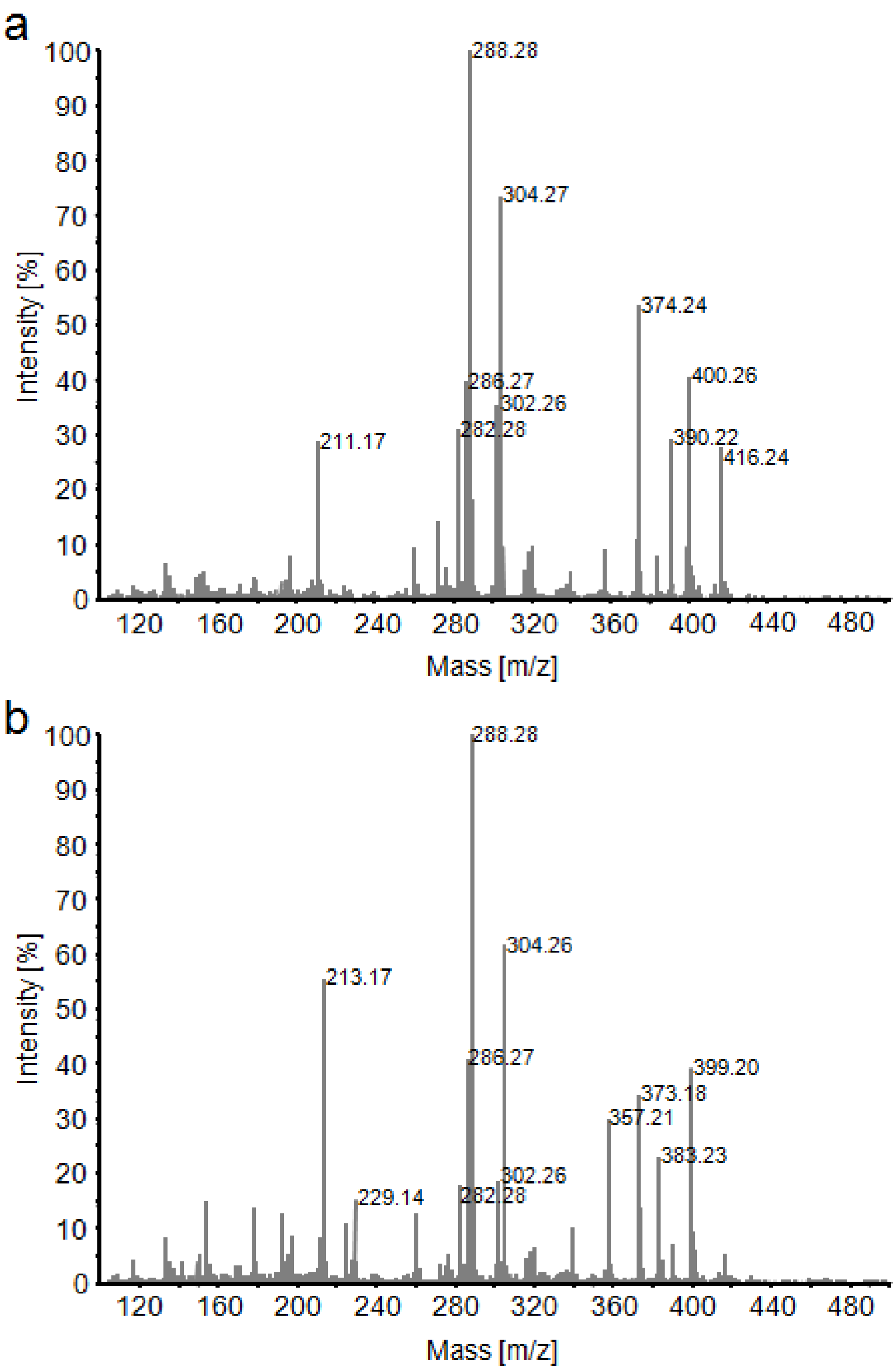

Figure S2: ESI-ToF mass spectrometry plot of purified foam extracts of *Rouxiella* sp. DSM 100043.

Lithium chloride supplemented subfractions (a) 63-65 C and (b) 63-65 D measured in positive mode**.** Both lithium $[M_d+Li^+]^+$ and sodium $[M_d+Na^+]^+$ adduct ions are present

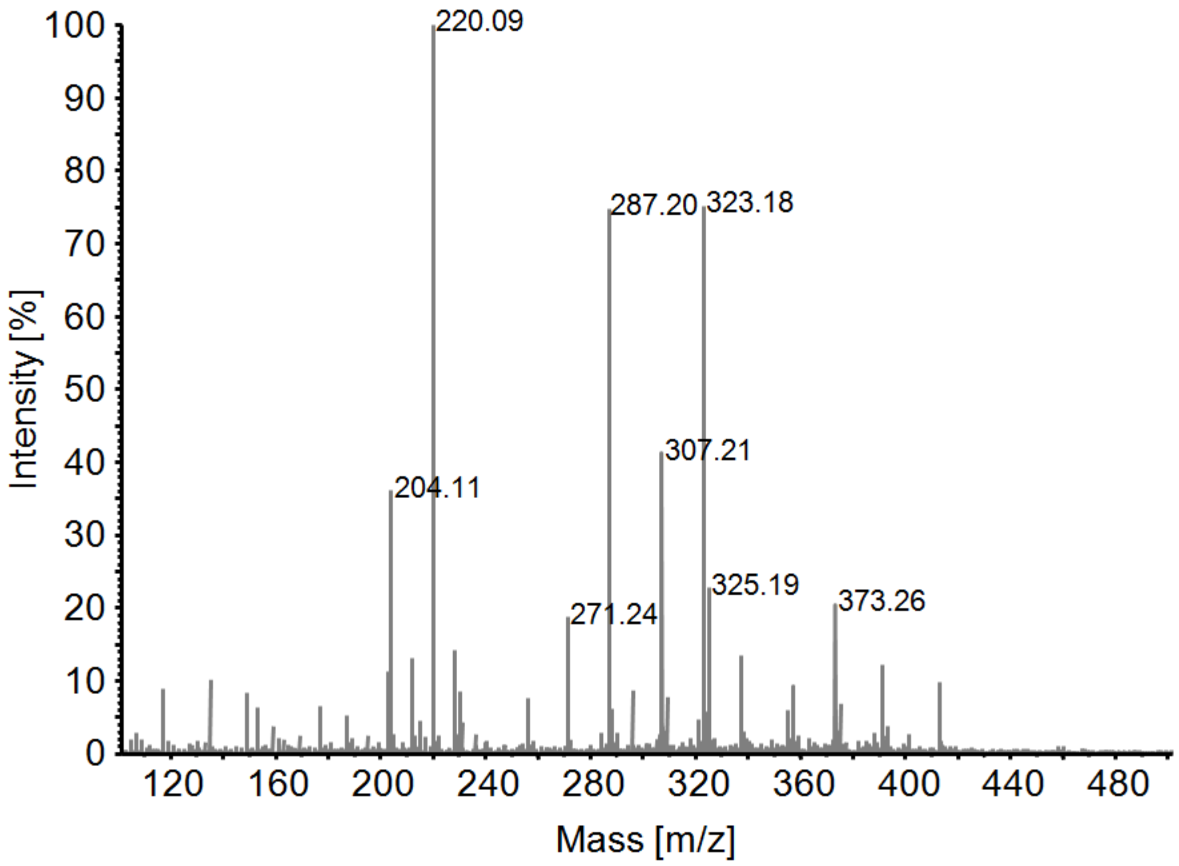

Figure S3: ESI-ToF mass spectrometry plot of purified foam extracts of *Rouxiella* sp. DSM 100043.

Lithium chloride supplemented subfraction 63-65 E measured in positive mode**.** Both lithium $[M_d+Li^+]^+$ and sodium $[M_d+Na^+]^+$ adduct ions are present

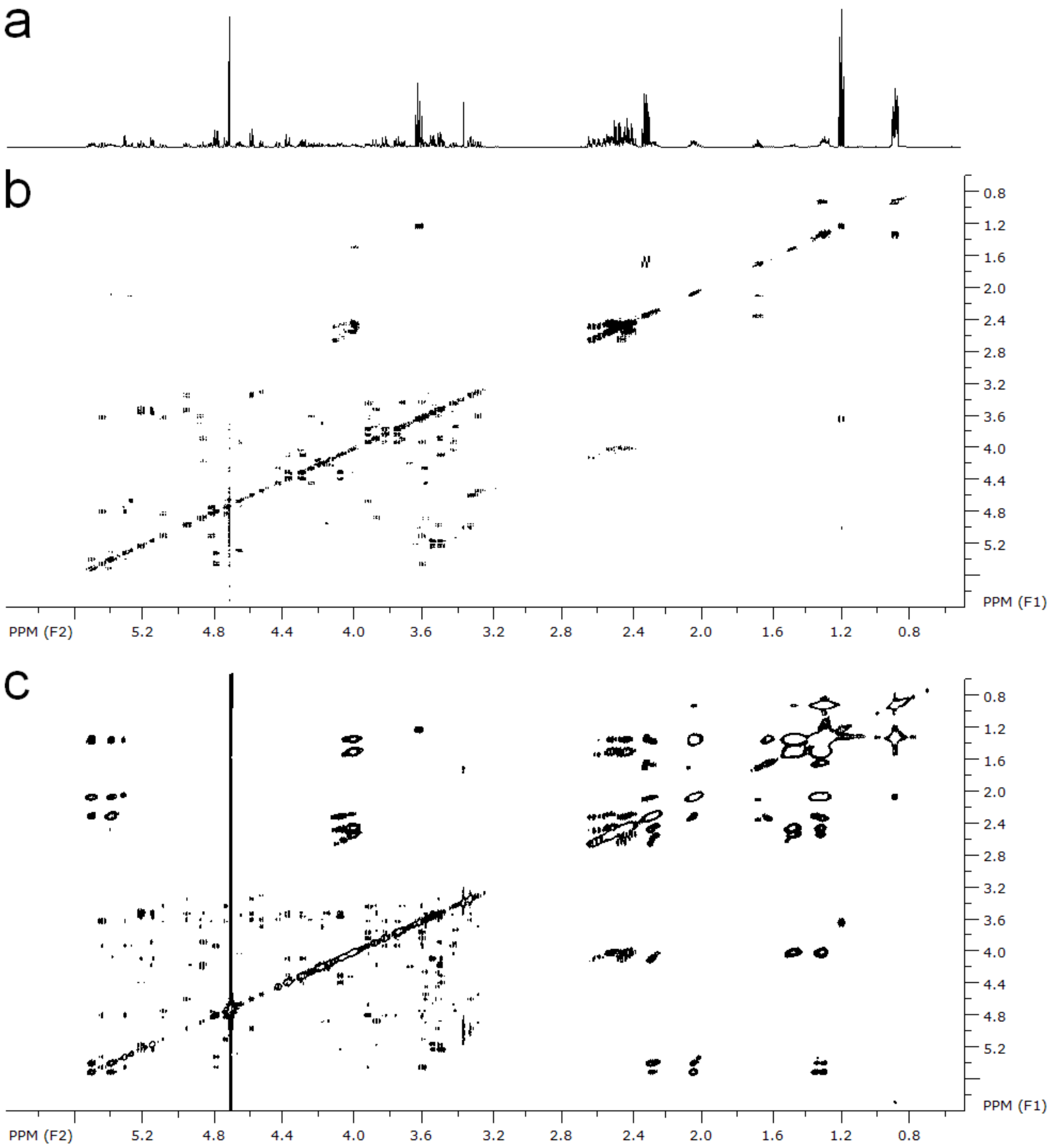

Figure S4: Full NMR spectra of *Rouxiella* sp. DSM 100043 glycolipids present in fractions 64-65.

(a) ^{1}H, (b) $^{1}H/^{1}H$ COSY and (c) $^{1}H/^{1}H$ TOCSY spectra recorded from fractions 64-65 after removal of residual fatty acids
